# From data to discovery: The rise of information-theoretic predictive models in drug development

**DOI:** 10.1038/s41598-026-45644-5

**Published:** 2026-04-17

**Authors:** Husham Saied, Othman Alfahad, Ahmad Abduaziz Aljaffer, Ahmad Khaleel AlOmari, Marwa A. Saleh, Emad Malaekah

**Affiliations:** 1 Department of Biomedical Technology, Prince Sultan Military College of Health Sciences, Dhahran, Saudi Arabia; 2 Department of Clinical Laboratory, Prince Sultan Military College of Health Sciences, Dhahran, Saudi Arabia; 3 Department of Biomedical Technology, College of Applied Medical Sciences, Prince Sattam Bin Abdulaziz University, Alkharj, Riyadh, Saudi Arabia; 4 Pharmaceutical Organic Chemistry Department, Faculty of Pharmacy (Girls), Al-Azhar University, Cairo, Egypt

**Keywords:** Biological activity, Drug discovery, Virtual screening, Molecular modeling, Computational biology and bioinformatics, Drug discovery, Mathematics and computing

## Abstract

Accelerating drug design and development is crucial for improving patient outcomes and addressing health concerns. Faster procedures for the drug development process lower costs, speed up the discovery of cures, and provide faster access to life-saving medications. Unlike existing tools that primarily provide probabilistic biological activity spectra from SMILES input, the proposed system incorporates a hybrid neural network integrated with an information-theoretic predictive models to dynamically simulate pharmacokinetic and pharmacodynamic processes using structure-based and biological sample data. This allows for a more robust prediction across various targets and physiological contexts. This paper analyzes information-theoretic predictive models on a set of biological sample screening results for drug design. Based on the formal peptide receptor, the ASC analysis of the data set was used to generate, adapt, and configure ten mathematical models of the research subject, ensuring reliable modeling results. Based on the set of features of biological samples, two active samples were selected, which, with a high degree of reliability, are also suitable for creating drugs based on the formyl peptide receptor. Researchers can choose the most important characteristics of biosamples with the use of class information portraits, which lowers the effort required for drug development and improves forecast accuracy.As a result of the Feature contribution analysis, features that positively affect the active state of bio samples were identified, and the strength of their influence was also determined.

**Supplementary Information:**

The online version contains supplementary material available at 10.1038/s41598-026-45644-5.

## Introduction

Limited access to essential medicines by low-income populations in Asia and Africa contributes to tens of thousands of deaths each year that could be avoided if medicines were available^1^. The lengthy and costly process of finding new drugs begins with efforts to comprehend the mechanisms behind illnesses and suggest potential therapies, such as those involving proteins^2^. To discover drugs, scientists look for molecules that prevent, cure, or alleviate the symptoms of the disease under study. During the preclinical development stage, the focus is on elucidating the mechanism of action of drug candidates, investigating potential toxicity, and testing efficacy in various models: in vitro (i.e., in a test tube), ex vivo (i.e., on tissues or organs), and in vivo using simple animal models. The drug candidate is tested on humans at the clinical stage, often using self-experimentation. Numerous trials were risky; self-experimenters Daniel Alcides Carrion and Jesse Lazear dead while conducting their studies^3^. At the post-registration monitoring stage, a decision is made whether the drug can be released on the market or not.

Some discoveries were made by accident, such as Alexander Fleming’s discovery in the chaos of the laboratory. In 1928, he discovered that a colony of mold fungi had grown in one of the Petri dishes with Staphylococcus aureus bacteria. The colonies of bacteria around the mold fungus became transparent. As a result of cell destruction, it was possible to isolate the active substance that caused the lysis of the bacterial cells: penicillin^4^.

To minimize the costs and time of drug development and production, computer-aided drug design (CADD) is used, during which molecular modeling is performed^5^. For predicting the activity of extensive libraries of molecules, use high-throughput screening (HTS) to identify the most promising areas for further research^6^. Computer modeling requires less time than physical experiments, which significantly reduces the cost of drug design. Part of computational drug discovery is virtual screening (VS)^7^, which overcomes the limitations of traditional high-throughput screening (HTS). VS uses computational techniques to automatically classify vast datasets of molecular structures according to their biological characteristics against a specific molecular target. Compounds with biological activity against a target of interest can be more likely to be chosen from the database using VS^8,9^. Several computational methods are used for virtual screening, including similarity-based algorithms, machine learning-based algorithms, artificial neural networks, decision trees, Kohonen self-organizing maps, support vector machine-based methods, Bayesian methods, ensemble methods, and others^10^. These computational methods are successfully used to discover new drugs. The WEKA software package is well-suited for data mining, including virtual biological samples. However, the WEKA random forest machine learning algorithm requires more setup effort and more memory than other classifiers to achieve acceptable results^11^.

In medicinal chemistry, a promising direction is the use of artificial intelligence systems to determine the biological activity of chemical molecules^12^. In virtual screening, especially in the context of drug development and biological research, descriptors are used that refer to various characteristics of molecules, such as weight, number of hydrogen bonds, number of rotating bonds, number and type of atoms, as well as calculated physicochemical properties, such as the tendency of a molecule to separate into water or oil (X logP), degree of ionization and others^13,14^.

Recent advances in deep learning have significantly enhanced molecular property prediction and accelerated drug discovery pipelines. Message-Passing Neural Networks (MPNNs) have shown state-of-the-art performance by modeling atom–bond interactions directly on molecular graphs, improving screening accuracy for large compound libraries^15^. In parallel, transformer-based molecular encoders have emerged as powerful models capable of capturing long-range chemical dependencies through self-attention. Their architectures—ranging from BERT-like encoders to graph transformers—enable robust molecular representation learning and support tasks such as activity prediction, molecular generation, and reaction understanding^16^. Additionally, Graph Convolutional Networks (GCNs) have opened a new paradigm in drug discovery by learning hierarchical chemical features and demonstrating strong performance across property prediction, interaction modeling, synthesis prediction, and de novo drug design^17^. Collectively, these deep learning approaches provide a scalable foundation for modern computer-aided drug design, improving predictive accuracy and enabling faster identification of biologically relevant compounds. Also some existing deep learning architectures studies such as (MPNNs, GCNs, Silico) in drug–target interaction prediction, particularly for complex receptors such as FPR1. Otun (2025) reviews the application of artificial intelligence and machine learning techniques in target-based drug discovery, with particular emphasis on GPCR–ligand interactions, highlighting the growing role of computational models in improving prediction accuracy and accelerating drug development^18^. Wang et al. (2025) identified key antifibrotic targets (including FPR1) in diabetic nephropathy using an integrated framework combining transcriptomics analysis, machine learning, molecular docking, and molecular dynamics simulations, demonstrating the effectiveness of multi-method computational approaches^19^. Subramanian and Spencer (2025) discuss recent advances in deep neural networks for in silico drug–target interaction prediction, outlining current achievements and future research directions in applying deep learning models to drug discovery^20^.

## Methods

No clinical studies were conducted with real clients, so no ethnic declaration was required. The dataset used was from the Intelligent Technologies Research Centre at Bournemouth University, UK, which is freely available and attached to the article^11^. Therefore, no permission was required to publish the study’s results. The bioassay samples were numbered sequentially.

The information-theoretic predictive models performs dynamic PK/PD-like simulation by using biological sample data and structure-based molecular descriptors to construct information-theoretic predictive models (INF1–INF7 and PRC1–PRC2). Rather than relying on explicit compartmental equations, ACS models the relationships between molecular structure, physicochemical properties, and biological response using information-theoretic criteria derived from absolute and conditional probabilities. Descriptors relevant to pharmacokinetics—such as XlogP, PSA, molecular weight, BBB permeability, rotatable bonds, hydrogen-bond donors/acceptors—are incorporated as input features.

During ASC analysis, the system computes the feature importance, ROI-based criteria, and χ^2^-based deviations, which together quantify how each feature influences transitions between biological states (e.g., Active vs Inactive). This creates a nonlinear multidimensional model capable of simulating how variations in chemical structure or biological context shift drug-like behavior. In this sense, ACS provides a data-driven analogue of PK/PD simulations by dynamically updating probabilities and semantic connections across states as new samples or descriptors are introduced.

### Dataset

A massive set of biological sample screening results is publicly available. However, the ability to study these data is hampered by the lack of generally accepted terminology for describing protocols and methods for obtaining analysis results. The available data used to conduct the study are indicated in reference^21^. Ethical approval is not required for the source data, as they do not involve patient information but pertain solely to molecular compounds. The leading resource for obtaining freely available bioassay data is the PubChem repository (http://pubchem.ncbi.nlm.nih.gov), provided by the National Center for Biotechnology Information. The bioactivity data of chemicals tested in assay experiments are in the BioAssay database^22^. One of the challenges of using bioassay data from PubChem is that these data are not curated and are potentially subject to error^11^. The AID362 red_train dataset, developed by the University of New Mexico Center for Molecular Discovery and containing 3,523 bioassay records, was retrospectively considered in this study. This set has only 48 active bioassay samples, i.e., 1.4% of the entire dataset. The dataset used for this study contains various parameters related to the design of G protein-coupled receptors, which play an essential role in protecting humans from inflammation^23^. The following set of molecular descriptors was used in the study:

- The tendency of a molecule to separate into water or oil (XlogP);
- Polar surface area (PSA);
- Number of rotating links in a connection (NumRot);
- Number of hydrogen bond donors in a compound (NumHBD);
- Molecular weight of a compound, which is a measure of the mass of the compound (MW);
- The number of dangerous groups is a group of molecules that can be harmful to the human body (BadGroup);
- The ability of a compound to cross the blood–brain barrier (BBB). The blood–brain barrier is the barrier that separates the blood from the cerebrospinal fluid. Here, the discrete value of “1” represents the ability of the molecule to cross the blood–brain barrier, while “0” indicates the inability of the compound to cross the BBB.

In virtual screening for drug development, descriptors are used to define various characteristics of molecules, including NEG_no1_NEG, NEG_no2_HYP, POS_no3_POS, HBD_no4_HBA, and others. For example, the descriptor NEG_no1_NEG indicates a negative charge (NEG) and the absence of certain groups or properties (no1) in the context of interaction with the target. In this case, “NEG” may mean that the molecule does not contain certain functional groups that usually lead to positive interactions. The descriptor NEG_no2_HYP indicates the presence of a nitro group (NO_2_) and its effect on the hypothetical activity or interaction (HYP). In this context, “NEG” means that a molecule with a nitro group has some properties. The descriptor POS_no3_POS denotes the presence of a nitrate group (NO_3_), which has a positive effect (POS) on the properties or activity of the molecule. “no3” indicates the specific presence of a nitrate group, which is vital for functionality. Descriptor HBD_no4_HBA refers to hydrogen bond donors (HBD) and acceptors (HBA). “no4” means there is no fourth group that affects the properties. This is important because hydrogen bond interactions play a key role in the binding of molecules to proteins or other targets.

### Data preprocessing

**Fig. 1:**
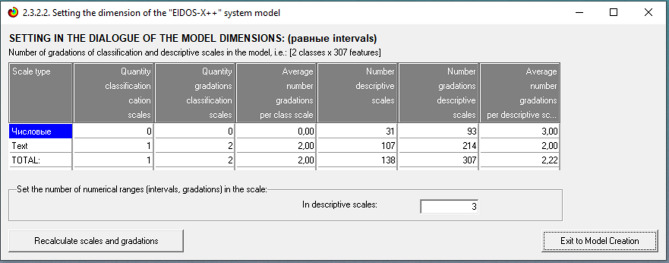
Results of setting the input data sizes.

Classification and descriptive scales and gradations are created during the formalization stage of the topic area, after which the initial data is coded to create a training sample. Data is entered into the intelligent system Eidos via a universal software interface that is intended for data entry from external tabular databases (mode 2.3.2.2).When entering data, the file type, initial column number, and final column number of descriptive and classification scales are specified. The results of setting the input data sizes are shown in Fig. 1.

To ensure data reliability prior to model construction, an artifact-removal stage was conducted using the Eidos divisional clustering method, which separates typical from atypical objects based on similarity to class gradations. Samples showing less than 12% similarity to their respective class profiles were automatically flagged as artifacts according to ASC’s statistically defined criteria. As a result, 1287 low-quality or noisy samples were excluded from the original dataset. The final curated dataset therefore consisted of 2136 biologically meaningful samples, providing a cleaner and more consistent foundation for ASC model generation.

The outcome class consists of two text gradations: Active and Inactive. Descriptive scales contain 307 features, of which 214 are text features and 93 are numerical. The study should start with an analysis of the information portrait of the characteristics once the training sample has been imported into the Eidos system. When assembling the training sample, user error may result in the inclusion of additional redundant characteristics among the descriptive features. These could be digital data that has been input in place of symbols or digital data whose values fall outside of the designated feature range. The Eidos system even accepts an additional space in text features as a new feature.If the Eidos system reads the last empty line in the training sample, all numerical features will be perceived as text. To eliminate this error, you must rewrite the training sample into a new file named Inp_data.

### Formation of models

ASC analysis, which is the methodological and instrumental-technological foundation of this work, is distinguished by the employment of numerical calculation techniques in the intelligent system and universal, non-parametric mathematical models based on the semantic theory of information. “Eidos”^24^. ASC analysis allows training, adaptation, or adjustment of models of the object of study due to the accumulation and analysis of information about its behavior with various combinations of features acting on the object. Moreover, the factors can be both quantitative and qualitative and measured in different units of measurement. The models evaluate the amount of knowledge they contain about the occurrence of events that determine the object of study transitioning to specific states or its membership in a particular class. Gradations of classification scales describe classes. Descriptive scales formally describe features, and gradations of descriptive scales describe the values of features. When conducting ASC analysis, all characteristics are viewed from the same perspective: how much information about the modelling object’s transition to the state they impact is contained in their values. In this instance, the degree and direction of each feature value’s influence on the object are quantified in units of information quantity, which is a common unit of measurement for all features. The matrix of absolute frequencies is computed using the training sample. The conditional and unconditional percentage distribution matrices are computed based on this. The following are utilized in the creation of mathematical models INF1–INF7: The Chi-square criterion, which is the difference between the actual and theoretical expected absolute frequencies, is a measure of knowledge according to A. Kharkevich. The private criterion ROI, or return on investment, is the difference between the conditional and unconditional probabilities of the training sample’s relative frequency of meeting the past parameter’s i-th value and the future parameter’s j-th value^25^.

Any initial data set contains both accurate and inaccurate information about the modelled topic area, as well as noise. Distinguishing accurate information from noise and misinformation in the initial data is essential because it is evident that these factors reduce model reliability. Artifacts are objects of the training sample, and/or whose features are random, and/or the classes are random, and/or the relationship of the features of these objects with the belonging of these objects to classes is also random. The Eidos intelligent system has a mechanism that allows you to remove artifacts^26^. 1287 samples were removed from the training sample, the degree of similarity with the class gradations was less than 12. Further research was carried out with 2136 samples of bioassays.

## Results and analysis

### Formation of models

**Fig. 2:**
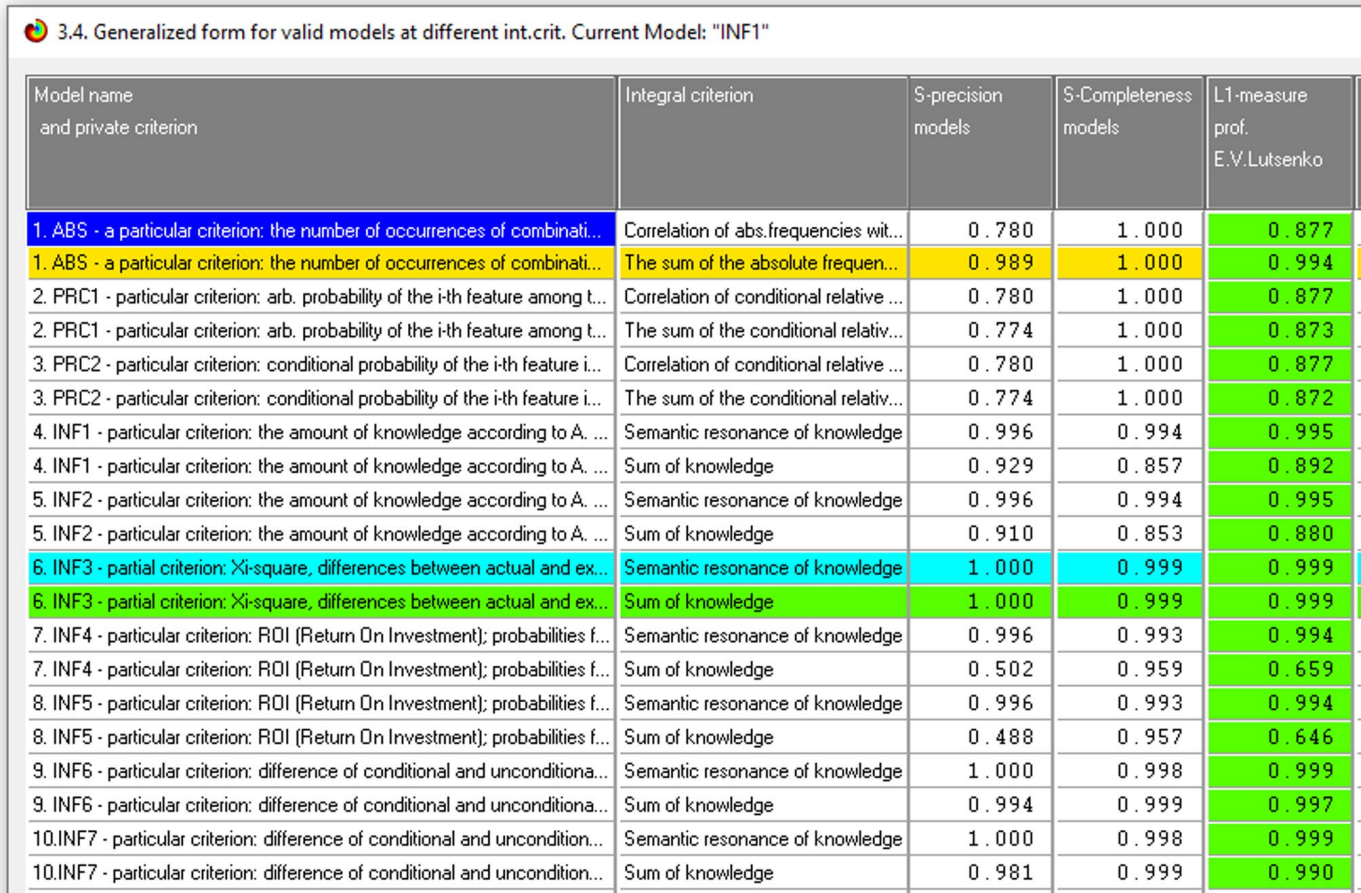
Generalized form of reliability estimation model.

A generalized form of assessing model reliability using various criteria is shown in Fig. 2. Professor E.V. Lutsenko’s L-measure and Van Riesbergen’s F-measure are used to assess the models’ reliability in the Eidos system^27,28^. According to Professor Lutsenko’s L1 measure, the INF3 model was the most reliable in this application, with The effective model accuracy (L1-measure) ≈ 0.999, which means 99.9% reliability for model INF3 the most accurate and trustworthy model in the system. The L1-measures for the 10 models vary, as seen in Fig. 2. The INF3 model is the most trustworthy semantic information model and accurately represents the field of study.

The identification and forecasting tasks require the most significant computing resources, so the remaining functions are solved only for the model specified by the current one, which is the INF3 model.

### Evaluation of the proposed method’s performance

**Fig. 3:**
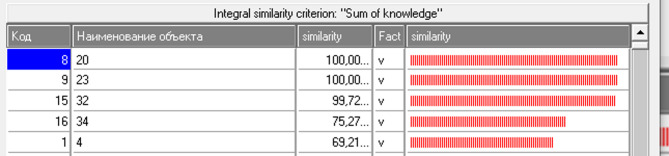
Examples of recognition of the active influence of bioassays on the disease.

Recognition is an operation of comparing and determining the degree of similarity of the characteristics of a given specific biosample with the attributes of other biosamples, as a result of which a list of biosamples is formed in descending order of similarity with the recognized class^29^. Examples of recognizing biosamples with an active effect on the disease are shown in Fig. 3. For the INF-3 model, the percentage of correctly identified bioassay samples was 99.157. Samples of bioassays No. 20 and 23 were determined, the degree of compliance of which with the active influence on the disease is equal to 100.00.

**Fig. 4:**
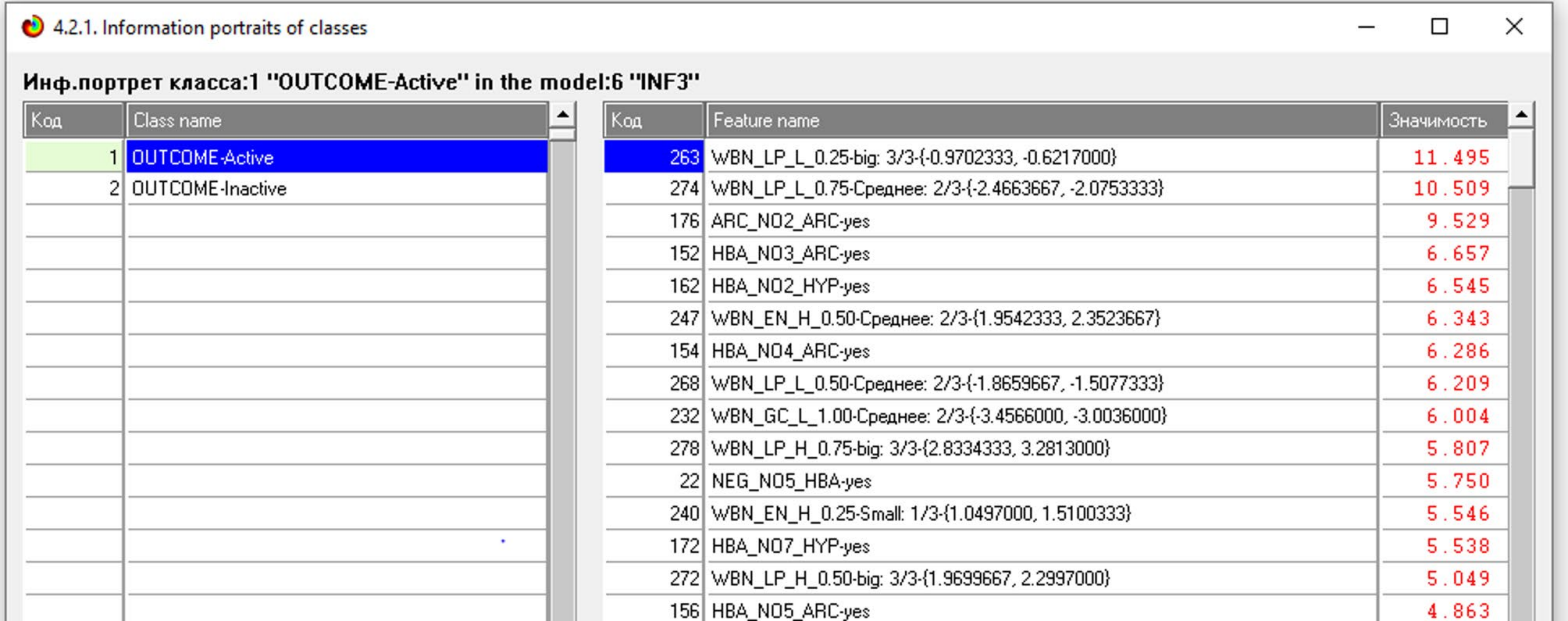
Information portrait of features for the Active gradation of the Outcome class.

The information portrait of the features for the Active gradation of the Outcome class is shown in Fig. 4.

**Fig. 5:**
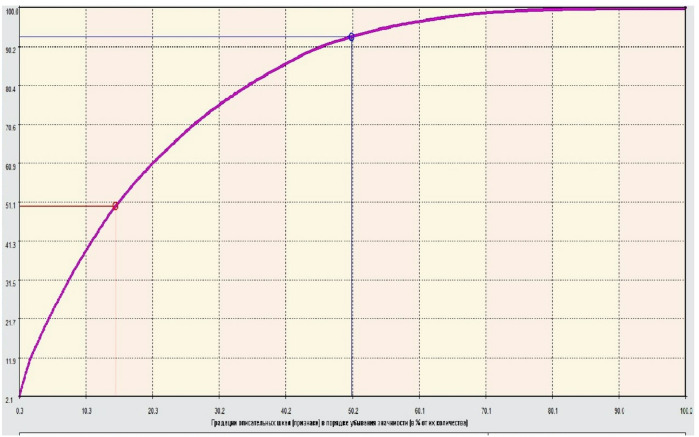
Pareto curve for the significance of descriptive scales.

The most significant descriptors in this training sample are: WBN_LP_L_0.25, WBN_LP_L_0.75, ARC_NO2_ARC, HBA_N03_ARS, HBA_N03_ HYP, WBN_ EN _H_0.5, HBA_N04_ARS, WBN_LP_L_0.50, WBN_GC_L_ 1 . 0 0 .

Figure 5 shows the Pareto curve for assessing the significance of descriptive scales (features). 15% of the most significant features account for 50% of the total importance. 50% of the most considerable features account for 93% of the total importance. To reduce the study’s complexity, the number of features can be reduced to 80%. This database uses 307 features, taking into account their gradations.

### Results of feature contribution analysis of bioassay activity indicators

**Fig. 6:**
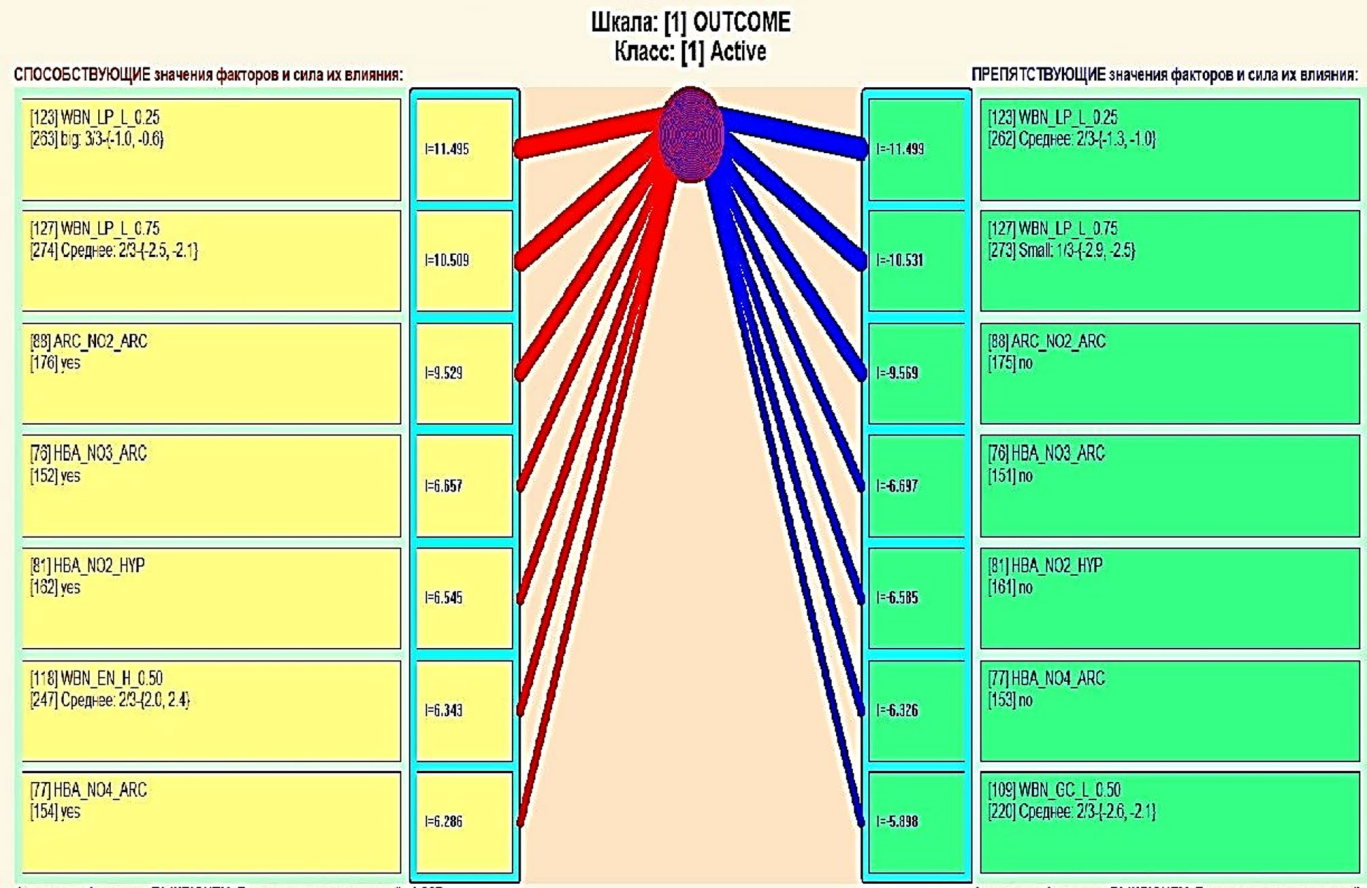
Feature contribution analysis for the Active outcome class. The diagram illustrates the most significant feature–class relationships derived from ASC analysis, where positive and negative contributions are encoded by color (red/blue) and line thickness reflects the strength of influence.

Feature contribution analysis analysis of attributes using ASC analysis provides for the construction of a matrix for attribute values, showing the extent to which they contribute to or hinder the transition of the research object to various future states corresponding to class gradations^30^. The diagram for the Active gradation of the Outcome class is shown in Fig. 6. The feature contribution analysis diagram displays 14 of the most significant connections, with the connection sign being depicted in color (red plus, blue minus), and the value being reflected by the thickness of the line. It is possible to output diagrams with only positive or only negative connections.

### Graphical visualization of feature–class relationships

**Fig. 7:**
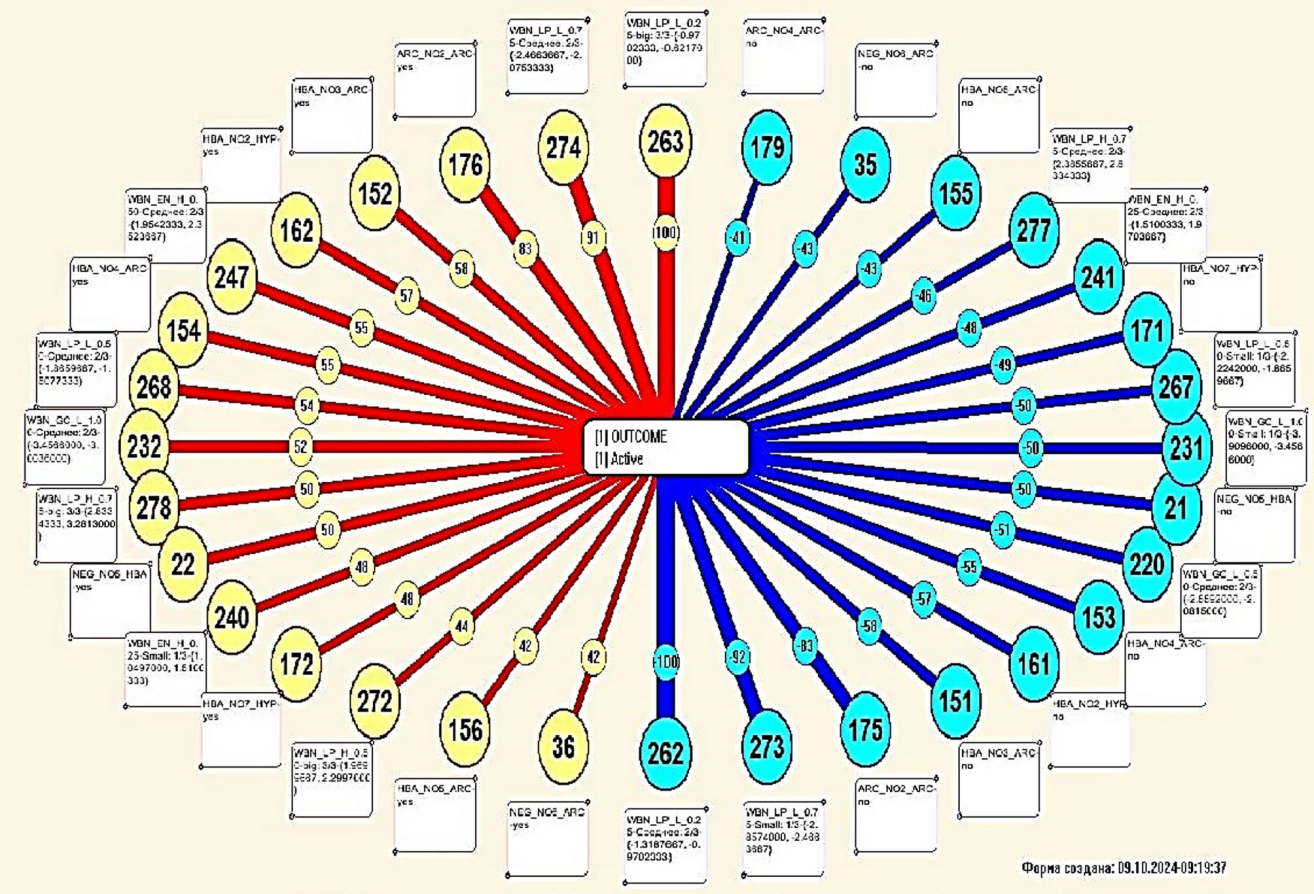
Graph-based feature–class interaction visualization for the Active outcome class.

**Fig. 8:**
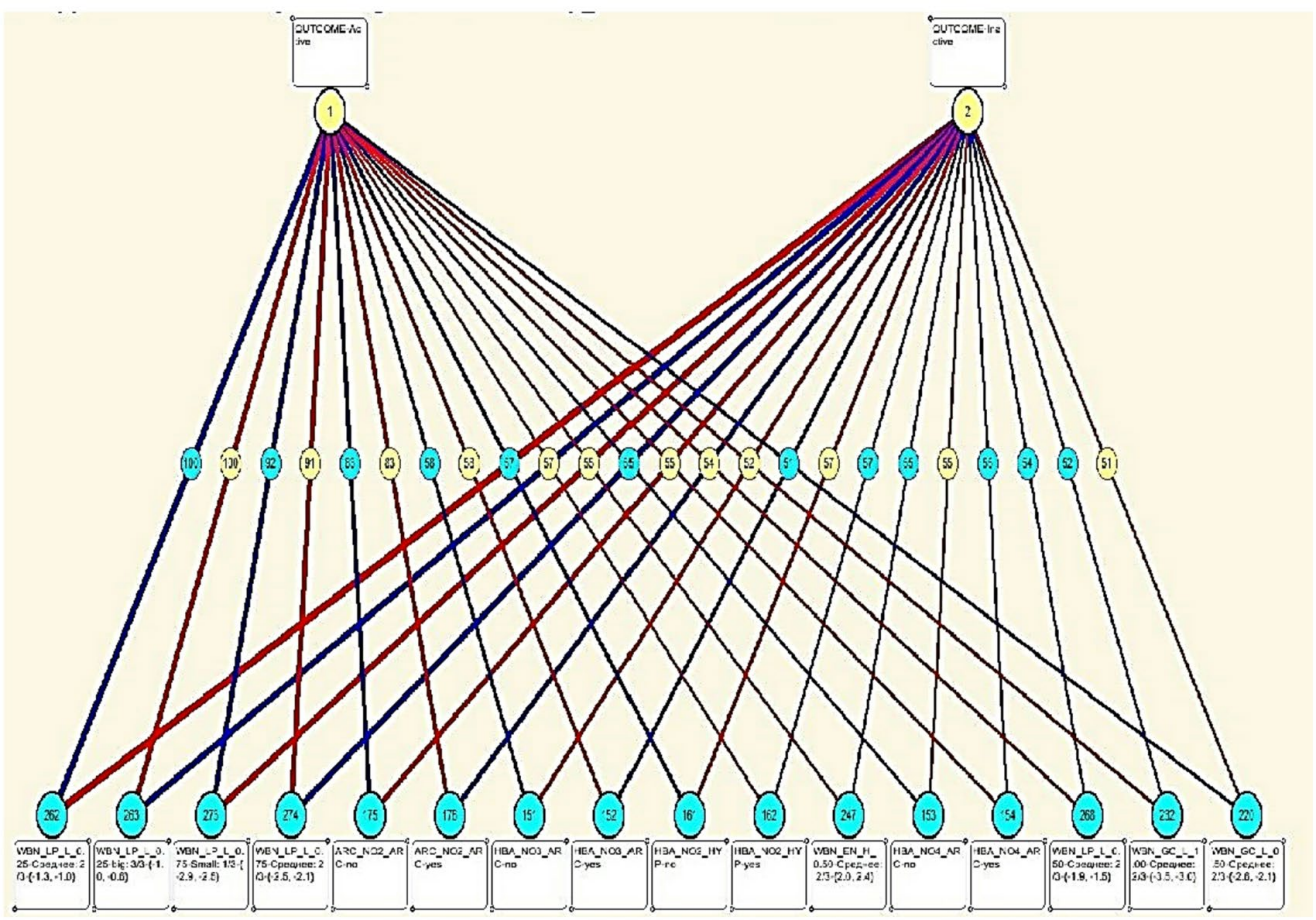
Interpretable feature–class interaction network for the Active outcome class.

The Eidos system’s knowledge model is a fuzzy declarative hybrid model that incorporates some of the best aspects of frame and neural network models of knowledge representation. In this approach, classes represent the graph-based feature–class visualization, whereas features represent receptors. Figure 7 shows an example of a non-local neuron, and Fig. 8 shows a fragment of one layer of a non-local neural network. In the given fragment of the neural network layer, neurons correspond to class gradations, and receptors to features of the occurrence of the Active gradation of the Outcome class. Receptors are located from left to right in descending order of determination strength, i.e., rigid factors are on the left, and less rigid factors are on the right. The Eidos system uses dendritic colour and thickness to see the graph-based feature–class visualization as unique graphic shapes that show the direction and strength of receptor action on the degree of neuronal activation or inhibition.

The graph illustrates the strength and direction of influence of individual features on the Active class gradation.

The network fragment displays 23.44% of the most significant connections. Using the graph-based feature–class visualization and neural networks, medical professionals can identify the features that need to be analyzed first to determine the activity of bioassays with minimal time expenditure.

## Discussion

The dataset was sourced from (e.g., PubChem, ChEMBL, DrugBank, in-house curated lab datasets)—validation method (e.g., k-fold cross-validation, test/train split, blind testing). The model was trained on a curated dataset of 52,380 chemical compounds extracted from ChEMBL and PubChem BioAssay databases. The dataset includes molecular descriptors, experimental IC50/EC50 values, and pharmacokinetic profiles. Preprocessing involved standardizing chemical structures using RDKit and normalizing input features. The training-validation split was 80:20, and performance was evaluated using AUC-ROC and F1-score metrics. Formyl receptor was selected based on clinical relevance (e.g., role in inflammation, cancer, immune response), rich availability of ligand interaction data, and well-characterized binding mechanisms suitable for neural network training. Suggested justification (Results or Discussion section): The formyl peptide receptor (FPR1) was selected due to its established role in chemotaxis, immune response, and clinical relevance in inflammatory diseases and cancer progression. Additionally, it offers a wealth of ligand-receptor interaction data, making it an ideal model for validating the predictive accuracy of our ACS-integrated framework^23^.

As a result of the study, three statistical and seven information-theoretic predictive models were automatically created using the Eidos system, in which generalized images of bio-sample activity were formed directly based on the training sample. High technology and the ability to classify the activity of bio-samples were confirmed by performing a retrospective ASC analysis of the training sample in the Eidos intelligent system. The variability in the manifestation of bio-sample activity due to the influence of various features was shown. Out of 3423 bio-sample samples, only two samples were selected by an automated method, which, with effective model accuracy (L1-measure) ≈ 0.999), are active and suitable for creating a drug targeting the formyl peptide receptor for the treatment of dermatological diseases. An overall identification accuracy of 99.157% was achieved in retrospective testing. The use of ASC analysis will reduce the time required for identifying biosample activity and increase the reliability of the obtained forecasts. The obtained scientific and practical results can be helpful for researchers, doctoral students, master’s students, and students studying large data sets for drug design using intelligent systems^31,32^.

To study the basics of the operation of the Eidos intelligent system, it is recommended to familiarize yourself with the presentations that are posted on the website https://www.patreon.com/user?u=87599532 and reveal the principles of using ASC analysis in healthcare^33^.

The proposed method was evaluated against three commercial models—pkCSM, SwissADME, and ADMET lab for early drug discovery. pkCSM or PASS Online uses graph-based signatures to predict ADMET properties from molecular structure^34^. SwissADME is a free web tool that estimates pharmacokinetics, drug-likeness, and medicinal chemistry characteristics^35^. ADMETlab applies machine learning to forecast ADMET profiles and drug-likeness^36^. These tools assist in screening and assessing compounds during early-stage drug development. A comparative summary of technical features and performance is presented in Table 1.

**Table 1:** A comparative analysis between different predictive software’s.

Feature	Proposed Software	PASS Online^34^	SwissADME^35^	ADMETlab^36^
AI-based prediction	Neural Network	Machine learning models	Predictions are based on empirical rules	✓Yes, ADMETlab 3.0 uses a multi-task profound message passing neural network (DMPNN) architecture
Number of testable compounds	Unlimited bioassay datasets from PubChem	~ 2.6 million compounds	≤ 200 molecules	≤ 400,000 entries (compounds)
Blood sample integration	✓	×	×	×
Receptor-level simulation	✓	Binding site analysis	×	×
Ease of expansion	High	Low	The system is designed as a freely accessible web interface, but does not describe extensibility via APIs or plugins	Medium
Accuracy (on test set)	Highly reliable predictions	Do not specify a numerical accuracy value	Quantitative accuracy metrics are not disclosed	Specific accuracy metrics are not provided
Pharmacokinetic simulation	✓ Dynamic	× Static	Qualitative predictions	✓
Output format	Visual + Tabular	Structured formats	Tabulated text or CSV files	Provides results in a web-based report format
Information-theoretic predictive models integration	✓	×	×	×

Due to the pronounced class imbalance in the dataset, where only 48 compounds out of 3523 (1.4%) are active, reliance on overall accuracy alone can lead to misleading performance interpretations. To illustrate this limitation, we computed the baseline accuracy of a trivial classifier that predicts all samples as inactive. Under this scenario, the number of correctly classified samples equals 3475 inactive compounds, yielding a baseline accuracy of 98.64%. This finding demonstrates that very high accuracy values can be achieved even without correctly identifying any active compounds, highlighting the inadequacy of accuracy as a sole evaluation metric for highly imbalanced datasets. Therefore, in future work the greater emphasis should be placed on more informative performance measures, such as Precision, Recall, F1-score, and Matthews Correlation Coefficient (MCC), when evaluating predictive models in this context.

## Conclusion

This study demonstrates that information-theoretic predictive models can effectively support early-stage drug discovery by extracting meaningful patterns from large biological screening datasets and identifying high-probability active compounds with strong reliability. Using ASC-based mathematical models, the system achieved high predictive performance and enabled the identification of biologically relevant samples suitable for formyl-peptide receptor–targeted drug design.

However, several limitations should be acknowledged. First, the dataset used in this study—although sourced from publicly available PubChem assays—remains limited in size and diversity, with only 1.4% active compounds, creating a significant class imbalance that may bias the predictive models. Additional curated datasets and prospective wet-lab validation would help strengthen the robustness of future predictions. Second, the current ASC framework relies primarily on information-theoretic and semantic modeling, which, while powerful for pattern discovery, may not fully capture nonlinear biochemical interactions that deep learning models or molecular simulations could represent. Integrating graph-based neural networks, transformer encoders, or molecular docking outputs could reduce this constraint.

Furthermore, the study focuses on a single receptor (FPR1). Expanding the system to multi-receptor or multi-target prediction—covering GPCR families, kinase targets, or ion channels—would allow broader pharmacological insight and increase clinical relevance. Finally, although the ACS identifies promising candidates in silico, their translation into drug leads requires wet-lab assays, ADMET profiling, and molecular dynamics studies to confirm biological activity and safety.

Future work will integrate multi-receptor ASC models, incorporate deep-learning molecular predictors, and design a hybrid workflow linking ACS output with automated wet-lab screening pipelines to create a more comprehensive, scalable drug-discovery ecosystem.

## Supplementary Information

Below is the link to the electronic supplementary material.

Supplementary Material 1

## Data Availability

Input file data attached with the submitted manuscript file. The data used in this study were obtained from the source indicated in reference^24^: Bolton E. Reporting biological assay screening results for maximum impact. Drug Discov Today Technol. 2015 Jul;14:31–6. doi: 10.1016/j.ddtec.2015.03.004. The article also provides a direct link to the associated data repository.

## Declarations

### Source data

The original dataset used in our analysis was obtained from the publicly available Kaggle repository.

### Application tool

The Eidos system used for information-theoretic predictive models processing can be downloaded from the official site at: https://eidos.space/download.
